# Voluntary saccade inhibition deficits correlate with extended white-matter cortico-basal atrophy in Huntington's disease

**DOI:** 10.1016/j.nicl.2017.06.007

**Published:** 2017-06-09

**Authors:** Israel Vaca-Palomares, Brian C. Coe, Donald C. Brien, Douglas P. Munoz, Juan Fernandez-Ruiz

**Affiliations:** a Centre for Neuroscience Studies, Queen's University, Kingston, ON, Canada; b Departamento de Fisiología, Facultad de Medicina, Universidad Nacional Autónoma de México, Mexico

**Keywords:** Saccades, Anti-saccade inhibition, Basal ganglia, Frontal cortex, White-matter atrophy, Bio-marker

## Abstract

The ability to inhibit automatic versus voluntary saccade commands in demanding situations can be impaired in neurodegenerative diseases such as Huntington's disease (HD). These deficits could result from disruptions in the interaction between basal ganglia and the saccade control system. To investigate voluntary oculomotor control deficits related to the cortico-basal circuitry, we evaluated early HD patients using an interleaved pro- and anti-saccade task that requires flexible executive control to generate either an automatic response (look at a peripheral visual stimulus) or a voluntary response (look away from the stimulus in the opposite direction). The impairments of HD patients in this task are mainly attributed to degeneration in the striatal medium spiny neurons leading to an over-activation of the indirect-pathway thorough the basal ganglia. However, some studies have proposed that damage outside the indirect-pathway also contribute to executive and saccade deficits. We used the interleaved pro- and anti-saccade task to study voluntary saccade inhibition deficits, Voxel-based morphometry and Tract-based spatial statistic to map cortico-basal ganglia circuitry atrophy in HD. HD patients had voluntary saccade inhibition control deficits, including increased regular-latency anti-saccade errors and increased anticipatory saccades. These deficits correlated with white-matter atrophy in the inferior fronto-occipital fasciculus, anterior thalamic radiation, anterior corona radiata and superior longitudinal fasciculus. These findings suggest that cortico-basal ganglia white-matter atrophy in HD, disrupts the normal connectivity in a network controlling voluntary saccade inhibitory behavior beyond the indirect-pathway. This suggests that in vivo measures of white-matter atrophy can be a reliable marker of the progression of cognitive deficits in HD.

## 1 Introduction

Inhibition of automatic motor actions in favor of more complex voluntary behavior in a demanding situation is a hallmark of executive functions for humans (Fuster, 2000). This ability can be impaired in different neurological conditions such as Huntington's disease (HD) (Anderson and MacAskill, 2013, MacAskill and Anderson, 2016, Watanabe and Munoz, 2011a), which is characterized by purposeless, involuntary and choreic movements (Walker, 2007). HD executive deficits could be the result of impairments in planning, flexible behavior, behavioral inhibition (Brandt et al., 1988, Brandt et al., 2008, Kassubek et al., 2005, Ross et al., 2014), and oculomotor control (Peltsch et al., 2008), the later probably due to disruptions in the interaction between basal ganglia and the saccade control system (Hikosaka et al., 2006, Munoz and Everling, 2004, Watanabe and Munoz, 2011a). These functional disabilities highlight the importance of characterizing inhibitory deficits for early diagnosis and disease progression in HD (Anderson and MacAskill, 2013, Ross et al., 2014).

Executive deficits can be studied with the interleaved pro- and anti-saccade task that requires flexible behavioral control to generate either automatic or voluntary movements according to specific task instructions (Munoz and Everling, 2004, Watanabe and Munoz, 2011a). The pro-saccade task requires an automated saccade to a peripheral visual stimulus, while the anti-saccade task requires the suppression of this automated response and instead the participant must look in the opposite direction. HD patients display inhibitory control deficits in this task, having increased and more variable saccade reaction times (Peltsch et al., 2008), and increased proportion of direction errors in the anti-saccade task, indicating a voluntary saccade inhibition deficit (Blekher et al., 2004, Lasker et al., 1987, Lasker et al., 1988, Patel et al., 2012, Peltsch et al., 2008, Scahill et al., 2013).

According to an influential basal ganglia model (DeLong, 1990), the inhibitory anti-saccade deficits in HD patients could be attributed to the degeneration of the striatal medium spiny neurons in the indirect-pathway through the basal ganglia arising from the putamen and caudate nuclei (Albin et al., 1990, DeLong, 1990). This degeneration leads to reduced inhibitory outflow from the internal globus pallidus/substantia nigra pars reticulata and excessive disinhibition of the thalamus that result in an excessive positive feedback to the motor areas (Albin et al., 1990, DeLong, 1990, Johnson et al., 2001, Sharp and Ross, 1996). Therefore, over-activation of the indirect-pathway disrupts the cortico-basal ganglia circuitry, decreasing the inhibition from the substantia nigra pars reticulata to the superior colliculus that leads to saccade suppression deficits (Hikosaka et al., 2006, Watanabe and Munoz, 2011b). However, the current basal ganglia model questions the indirect-pathway hypothesis suggesting instead an interaction between direct, indirect and hyperdirect-pathways in executive-motor control (Jahanshahi et al., 2015, Nambu et al., 2002). In this new model, signals from supplementary motor area, premotor, prefrontal, parietal cortices and cingulate gyrus are first transmitted through the hyperdirect-pathway, induce early excitation in the substantia nigra reticulata, and inhibit inappropriate movements; then signals through the direct-pathway inhibit the substantia nigra reticulata and release appropriate movements; and finally, signals through indirect-pathway induce late excitation in the substantia nigra reticulata and stop movements (Nambu et al., 2002). Thus, it is possible that degeneration in other brain areas beyond the indirect-pathway (Desmurget and Turner, 2008, Kassubek et al., 2005, Klöppel et al., 2008, MacAskill and Anderson, 2016, Nambu et al., 2002, Okun and Vitek, 2004, Sano et al., 2013, Vintonyak et al., 2017, Watanabe and Munoz, 2011a, Zhuang et al., 2009) and their interconnectivity (Gorges et al., 2016, Matsui et al., 2015), could contribute to the executive and saccade deficits in HD. One way to delve into this debate is by analyzing anti-saccade (Munoz and Everling, 2004, Watanabe and Munoz, 2011a) and anticipatory behaviors in oculomotor tasks that probe inhibitory control (Spengler et al., 2006), as well as structural magnetic resonance imaging to map cortico-basal atrophy in HD patients.

We test the hypothesis that atrophy in hyperdirect-pathway related areas including frontal; parietal, cingulate cortices and with-matter connecting them, are related with voluntary oculomotor control deficits in early HD patients. To this end we evaluated the brain atrophy in HD using Voxel-based morphometry, Tract-based spatial statistic and its effect on voluntary oculomotor control in the interleaved pro- and anti-saccade task. We were especially interested in anti-saccade errors and anticipatory saccades as a measure of voluntary oculomotor control and its relationship with neural atrophy. Our results suggest that an extended neural network beyond the indirect-pathway mediates voluntary saccade inhibition control in HD.

## 2 Methods

### 2.1 Participants

The Queen's University and Universidad Nacional Autónoma de México Health Science and Ethics Committees in Human Research approved all experimental procedures in accordance with declaration of Helsinki (World Medical Association, 2013). All HD patients and control participants provided informed consent, and were recruited and evaluated at the Instituto Nacional de Neurología y Neurocirugía (INNN) México. Only HD patients with molecular diagnosis of the CAG trinucleotide repeat expansion (CAG) and whose neurological/motor impairment did not prevent performing the test were included. The HD group consisted of 23, right-handed patients aged 29–68 years, 13 females; mean ± SD age 49.6 ± 11.4; age at onset 25–62 years, mean 44.6 ± 10.1; early disease duration from 1 to 10 years, mean 4.5 ± 2.9; repeats size CAG 40-52, mean 44.2 ± 3.0; and years of education 9–18 years, mean 13.5 ± 3.0 see Table 1 for details. HD patients did not take any specific medication for the disease; they only were given supplements such as Coenzyme Q-10, and were not asked to interrupt it during the recording session. We exclude one patient due to extensive eye tracking loss. The control group consisted of 23 healthy right-handed volunteers (age 30–64 years, 13 females, mean age 49.9 ± 10.6; and years of education 9–20 years, mean 15.9 ± 3.3) that were age- and sex-matched to the HD patients. Control participants did not report any visual, neurological or psychiatric disorder and had Montreal Cognitive Assessment (MOCA) scores ≥ 24, mean 27.2 ± 1.6 (see Table 1) as assessed by the experimenter IVP.

**Table 1:** Summary of demographic and clinical information.

Group	Age	Gender male/female	Education	MOCA	Age at onset	CAG	UHDRSm	UHDRSf	DD
CTRL	49.9 ± 10.6	10/13	15.9 ± 3.3	27.2 ± 1.6					
HD	49.6 ± 11.4	10/13	13.5 ± 3.0	24.1 ± 3.3	44.6 ± 10.1	44.2 ± 3.0	17.5 ± 12.5	11.3 ± 2.2	4.5 ± 2.9

An expert neurologist from INNN performed clinical evaluation for HD patients. The evaluation included the MOCA to assess the general cognitive functioning and the Unified Huntington's Disease Rating Scale (UHDRS) to assess disease progression (‘Unified Huntington's Disease Rating Scale’, 1996). HD patients scored a mean of 24.1 ± 3.3 in the MOCA, and a mean of 17.5 ± 12.5 on the motor and 11.3 ± 2.2 on the functional components of the UHDRS (Table 1). It should be noted that the motor component of the UHDRS has standardized ratings of oculomotor function, dysarthria, chorea, dystonia, gait and postural stability.

### 2.2 Experimental procedure and paradigm

Right-eye position was recorded in all participants (patients and controls) with a video based eye tracker (Eyelink-1000 Plus monocular-arm; SR Research, Mississauga, ON, Canada) at a rate of 500 Hz with monocular recording. Stimulus presentation and data acquisition were controlled by Eyelink Experiment Builder and EYELINK software. Participants were seated in a dark room, while seated the participants comfortably rested their heads in a sturdy chin and forehead support avoiding head movements as much as possible. The stimuli were presented on a 17-inch LCD monitor at a screen resolution of 1280 × 1024 pixels (60-Hz refresh rate), subtending a viewing angle of 32° × 26°, and the distance from the monitor to the eyes was 60 cm.

**Fig. 1:**
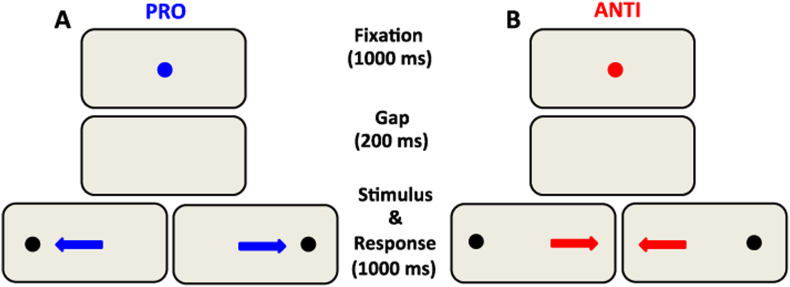
Paradigm representation of the stimulus and timing for the two trial conditions. Each trial started with a central colored fixation point; two isoluminant colors for the pro-saccade A) and anti-saccade B) trials on a dark background. After 1000 ms a blank screen was presented for 200 ms (gap) before stimulus presentation. Note that the fixation point colors displayed here are only for illustration. Participants were required to move their eyes to the stimulus in the pro-saccade trials, but move to the opposite direction in the anti-saccade trials. Arrows indicate the correct saccade directions on each trial and were not actually displayed.

The eye movement experiment consisted of pseudorandomly interleaved pro- and anti-saccade trials. The participants completed two blocks of 120 trials lasting approximately 7 min each. Each trial started with the presentation of a central fixation point (FP) (diameter, 0.5°; 42 cd/m^2^) on a black background (0.1 cd/m^2^). The trial condition was revealed via the FP color, green for pro-saccade instruction and red for anti-saccade instruction. After 1000 ms, the central FP disappeared for 200 ms before the peripheral stimulus appeared (diameter, 0.5°; white dot with luminance 42 cd/m^2^) to the left or right of the FP (10° eccentricity on the horizontal axis) for 1000 ms (Fig. 1). Introducing a gap period between FP disappearance and stimulus appearance forces disengagement of active fixation before target appearance and leads to reduction in reaction times for both pro- and anti-saccades and to an increase in direction errors in the anti-saccade trials (Munoz et al., 1998). In the pro-saccade trials, the participant had to make a simple automatic saccade to a peripheral stimulus, whereas in the anti-saccade trials the participant had to suppress the automatic saccade and made a voluntary saccade to the opposite direction instead (Fig. 1). The task was preceded by a short practice period. The instructions were repeated before each experimental block began. Trial condition (pro-saccade and anti-saccade) and stimulus location (left or right) were randomly interleaved. Saccades toward either the right or left direction were combined for data analysis. Every participant corrected a number of errors by making a secondary saccade to the correct location, which confirmed their understanding of task instruction.

### 2.3 Behavioral data analysis

The onset and termination of each saccade was determined when the eye velocity exceeded 30°/s. Trials were scored as correct if the first saccade after stimulus appearance was in the correct direction (toward the stimulus in the pro-saccade trials; and away from the stimulus in the anti-saccade trials). Responses were classified as direction errors if the first saccade after the stimulus appearance was in the wrong direction. Saccadic reaction time (SRT) was measured from stimulus appearance to onset of the first saccade. Saccades initiated prior to when visual stimulus information was able to reach the oculomotor brain regions (i.e., SRT < 90 ms) were categorized as anticipatory saccades (Bell et al., 2006, Marino et al., 2012), because they were equally likely to be correct or incorrect. Short-latency stimulus-driven saccades within the first peak of a multimodal distribution of SRTs were identified as express saccades (Fischer et al., 1984). We used anti-saccade direction error latencies in combination with correct pro-saccade latencies to help identify the short latency saccade epoch. A binomial sign test determined the start and end of the short-latency saccade epoch by measuring when the proportion of anti-saccade error trials (in 10-ms bins) significantly exceeded that of correct anti-saccade trials. Saccades with SRTs between 90 and 140 ms were identified as express saccades, SRTs > 140 ms were identified as regular latency saccades. We computed the following values, for each subject pro- and anti-saccade trials with latencies from 90 to 1000 ms: mean SRT and coefficient of variation of SRT (standard deviation/mean × 100%) for all correct saccades. Note that the difference between anti- and pro-saccade reaction times, known as the anti-effect, provides a measure of the time it takes for additional anti-saccade processes-inhibition of a stimulus driven saccade and the voluntary initiation of the correct anti-saccade (Munoz and Everling, 2004). We also calculated the percentage of anticipatory saccades (SRT < 90 ms); percentage of express saccades (SRT = 90–140 ms); percentage of regular-latency saccades (SRT > 140 ms); percentage of direction errors (erroneous pro-saccades to the stimulus in the anti-saccade trials) in both express- and regular-latency saccades (Coe and Munoz, 2017). All behavioral data were analyzed using custom written scripts in MATLAB-R2014a. Before any statistical analysis, the appropriate normality test (Shapiro-Wilk) was made for all variables.

Our data showed statistical differences between HD patients and controls in terms of years of education (*t*_(44)_ = 2.475, *p* = 0.017) and MOCA scores (*t*_(44)_ = 3.933, *p* = 0.000). To find out if there was a relationship between saccade measures and these variables influencing the results, we compute the correlations in terms of direction error proportion, error proportion in express-latency, error proportion in regular-latency, SRTs, proportion of anticipatory saccades and coefficient of variation of SRT. All correlations were found to be not significant (data not shown).

### 2.4 Imaging protocol acquisition

All images were acquired using a 3.0 Tesla Achieva MRI (Philips Medical System, Eindhoven, Holland) scanner with a 32-channel head coil. The study included the acquisition of a high-resolution T1 three-dimensional (3D) volume and Diffusion-weighted imaging (DWI). The T1-3D acquisition consisted of a T1 Fast Field-Echo sequence, with repetition time/echo time (TR/TE) = 8/3.7 ms, field of view (FOV) 256 × 256mm^2^; and an acquisition and reconstruction matrix of 256 × 256, resulting in an isometric resolution of 1 × 1 × 1mm^3^. The DWI sequence consisted of a single shot echo planar Imaging sequence acquiring 33 volumes of 70 axial slices (2 mm slices thickness and no separation), one of each of 32 independent directions of diffusion with b = 800 s/mm^2^ and one corresponding to b = 0 s/mm^2^, TR/TE 8467/60 ms, FOV 256 × 256mm^2^ and an acquisition and reconstruction matrix of 128 × 128, resulting in an isometric resolution of 2 × 2 × 2mm^3^.

### 2.5 Imaging analysis

#### 2.5.1 Grey-matter

T1 images were analyzed through the Voxel-based morphometry technique implemented on FMRIB Software Library (FSL) (http://fsl.fmrib.ox.ac.uk/fsl/fslwiki/FSLVBM). First, voxels that did not belong to the images of the cerebral tissue were eliminated using (Brain Extraction Tool) BET software. Afterwards, segmentation was done according to the type of tissue sampled (grey-matter, white-matter and cerebrospinal fluid) using FAST4. The images corresponding to the grey-matter were aligned to the MNI152 standard space by means of a linear followed by a non-linear co-registration in order to obtain optimal co-registration. The average of these co-registered images was obtained to generate the specific standard for this study. The grey-matter images were co-registered to this specific standard through a non-linear co-registration and local changes in expansion or contraction were corrected by modulation. A smoothing process was applied with a Gaussian isotropic kernel with a sigma of 4 mm. An independent two-sample *t*-test was applied through the general linear model correcting for multiple comparisons using randomized permutation methods (n = 10,000). We chose the appropriate smoothing (sigma 4 mm) for the Threshold-Free Cluster Enhancement (TFCE) based analysis. The family-wise error (FWE) was controlled and only corrected 1 – *p* values ≤ 0.095 were accepted.

#### 2.5.2 White-matter

DWI images were processed using the FSL's Diffusion Toolbox (http://fsl.fmrib.ox.ac.uk/fsl/fslwiki/TBSS). Eddy current effects were corrected by taking the first volume of the sequence as a reference. The diffusion tensor model was adjusted to generate the fractional anisotropy (FA) images for each subject. The statistical analysis was done in voxel-wise manner using the Tract-Based Spatial Statistic methodology. First, FA images were created by fitting a tensor model to the raw diffusion data and then brain-extracted using BET. All subjects' FA maps were aligned into a common space using a non-linear registration tool FNIRT, which uses a b-split representation of the registration warp field. Next, the mean FA image was created and thinned to create a mean FA skeleton that represents the center of all tracts common to the group. Each subject's aligned FA was then projected into this skeleton and the resulting data fed into voxel-wise cross-subject statistic. An independent two-sample *t*-test was applied through the general linear model correcting for multiple comparisons using randomise permutation methods (n = 10,000). The only voxels considered as showing a significant difference between groups were those with *p* < 0.05, corrected.

In a third analysis, we tested if there was a relationship between grey-matter volume and/or white-matter FA and performance on the saccade variables. We performed a one–sample *t-*test in a voxel-wise analysis through the general linear model correcting for multiple comparisons using randomise permutation methods (n = 10,000) of the HD patients for grey-matter volume and white-matter FA independently. Age and CAG repeat lengths are known to influence the onset and trajectory of the disease and Disease-burden score was calculated (age × [CAG repeat length – 35.5]) to represent an interaction between these two terms, and included as covariates in the model (Scahill et al., 2013). The only voxels considered as showing a significant difference were those with *p* < 0.05.

### 2.6 Statistical analysis

We conducted Mixed-design ANOVAs (between-subjects factor: Huntington's disease/age-matched control × within-subject factor: anti/pro-saccade) to examine differences in behavior between groups in terms of direction error proportion, error proportion in express-latency, error proportion in regular-latency, SRT, proportion of anticipatory saccades and coefficient of variation of SRT. Non-parametric two-sample Kolmogorov-Smirnov test were conducted to compare the SRT cumulative distributions between the groups. Correlations between anti-saccade direction errors and anticipatory anti-saccades were computed by the two-tailed Pearson's correlation coefficient.

## 3 Results

### 3.1 Saccade behavior

#### 3.1.1 Distribution of SRTs

**Fig. 2:**
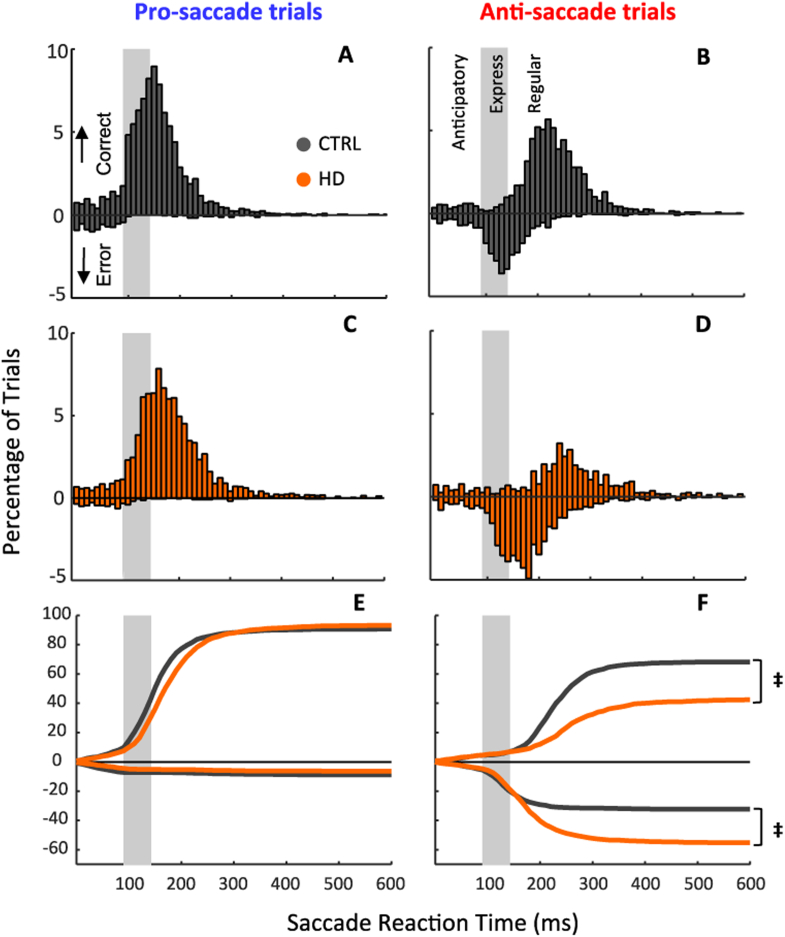
Instantaneous and cumulative histograms for SRT. Distributions of SRTs for the pro-saccade trials of A) control (CTRL) and C) Huntington's (HD) groups. Distributions of SRTs for the anti-saccade trials of B) CRTL and D) HD groups. Positive ordinate values indicate correct saccades, whereas negative ordinate values indicate direction errors for saccades initiated between 0 and 600 ms. However, saccades between 0 and 90 ms were considered anticipatory regardless of direction. Grey shaded boxes represent the range of calculated express saccades (90–140 ms) (see Methods). E) Cumulative distribution for pro-saccade SRTs for both CTRL (dark grey) and HD (orange) groups. F) Cumulative distribution in anti-saccade trials for both CRTL (dark grey) and HD (orange) groups. ‡ indicates significant difference (*p* = 0.001). (For interpretation of the references to color in this figure legend, the reader is referred to the web version of this article.)

Fig. 2 shows the distribution of SRTs for correct responses (positive values on ordinate) and direction errors (negative values on ordinate) for all participants in each group for pro- and anti-saccade tasks. The distributions highlight 3 discrete epochs: anticipatory (SRT < 90 ms), express (SRT = 90–140 ms), and regular (SRT > 140 ms) latency responses. The proportion of anticipatory saccades was similar for both controls and HD patients, but the error proportion in regular-latency were higher for HD patients in the anti-saccade task. In general, during the pro-saccade task (Fig. 2E) the control group was slightly faster than HD patients. In contrast, during the anti-saccade task (Fig. 2F), the controls responded much faster than HD patients. The analysis of the SRT cumulative distribution with a non-parametric test (two-sample Kolmogorov-Smirnov) revealed that pro-saccade SRT distributions did not show statistical differences between controls and HD patients for correct (*K* = 0.411 *p* = 0.996) and erroneous (*K* = 0.411 *p* = 0.996) trials (E). However, anti-saccade SRT distributions between groups showed significant differences in correct (*K* = 2.008 *p* = 0.001) and erroneous (*K* = 2.008 *p* = 0.001) trials (F).

**Fig. 3:**
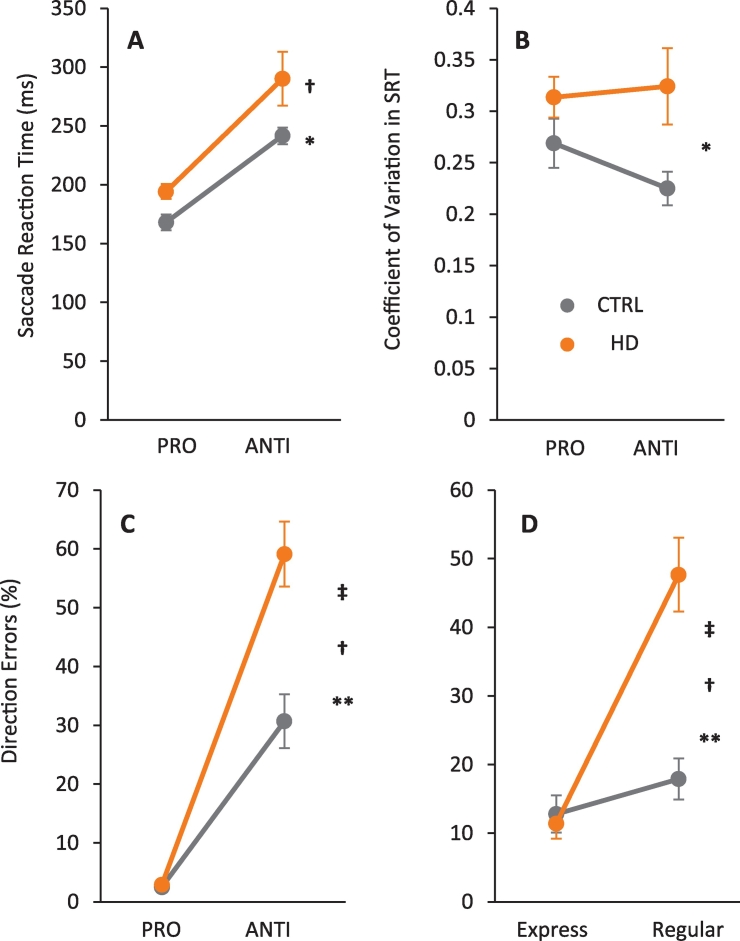
Saccade behavior for both CTRL and HD groups in pro- and anti-saccade trials. A) Mean proportion of saccade reaction times for all correct saccades. B) Mean intrasubject variability for all correct saccades. C) Mean percentage of direction errors. D) Mean percentage of direction errors saccades that were express-latency (Express) or regular-latency (Regular). Error bars indicate SEM. (†) *p* = 0.000, significance for task interaction only. (*) *p* = 0.012, significance for group interaction only (saccade reaction time). (*) *p* = 0.009, significance for group interaction only (intrasubject variability). (‡) *p* = 0.000, significance for group × task interaction only. (**) *p* = 0.000, significance for group interaction only.

To contrast performance between subject groups, we calculated the mean SRT for all correct responses (SRT > 90 ms) for each subject for the pro- and anti-saccade tasks. The ANOVA analysis showed a main effect of task (*F*_(1,43)_ = 62.268, *p* = 0.000, *η*^2^_*p*_ = 0.592) with significantly prolonged mean anti-SRT (anti-effect (SRT-anti – SRT-pro)). The analysis also found a main effect of group (*F*_(1,43)_ = 6.825, *p* = 0.012, *η*^2^_*p*_ = 0.137) where the HD patients had a larger mean anti-saccade SRT than the controls. However, there was no main effect of group × task interaction (*F*_(1,43)_ = 1.085, *p* = 0.304, *η*^2^_*p*_ = 0.025) (Fig. 3A). We also examined intra-subject variability in SRT, expressed as the coefficient of variation (Fig. 3B). The analysis showed a main effect of group (*F*_(1,43)_ = 7.426, *p* = 0.009, *η*^2^_*p*_ = 0.147) where HD patients had more variability in SRT than controls. However, there were no main effects for task (*F*_(1,43)_ = 0.471, *p* = 0.496, *η*^2^_*p*_ = 0.011) or group × task interaction (*F*_(1,43)_ = 1.257, *p* = 0.268, *η*^2^_*p*_ = 0.028).

#### 3.1.2 Saccade direction errors

The proportion of direction errors in anti-saccade trials provides a robust measure of inhibitory control (Munoz and Everling, 2004). We computed the proportion of direction errors for pro- and anti-saccade trials. The analysis of group by condition interaction resulted in significant main effect of task (*F*_(1,43)_ = 133.741, *p* = 0.000, *η*^2^_*p*_ = 0.757). More errors occurred in the anti-saccade trials; a main effect of group (*F*_(1,43)_ = 16.505, *p* = 0.000, *η*^2^_*p*_ = 0.277) indicated that the HD patients made more errors; and the main effect of group × task interaction (*F*_(1,43)_ = 14.725, *p* = 0.000, *η*^2^_*p*_ = 0.255) revealed an increase in direction error proportion for anti-saccade due to patients' errors (Fig. 3C). We also computed the proportion of errors that occurred in the express- (SRT = 90–140 ms) and regular-latency epochs (SRT > 140 ms) in the anti-saccade task to determine if the increased error rate in HD patients was due to a selective increase in errors with faster or slower SRT (see Table 2). The analysis showed a significant main effect of task (*F*_(1,43)_ = 35.738, *p* = 0.000, *η*^2^_*p*_ = 0.457), due to the larger proportion of regular-latency errors; a main effect of group (*F*_(1,43)_ = 15.773, *p* = 0.000, *η*^2^_*p*_ = 0.321) due to the larger number of errors in HD patients; and a main effect of group × task interaction (*F*_(1,43)_ = 20.288, *p* = 0.000, *η*^2^_*p*_ = 0.321) showing that the HD patients made more errors in regular-latency epoch (see Table 2) (Fig. 3D).

**Table 2:** Main results in anti-saccade condition.

Group	Saccade direction errors		Anticipatory saccades
	Express	Regular	
CTRL	12.8 ± 12.9	17.8 ± 14.2	9.2 ± 6.9
HD	11.4 ± 10.3	47.6 ± 25.3	16.1 ± 13.9

#### 3.1.3 Anticipatory saccades

**Fig. 4:**
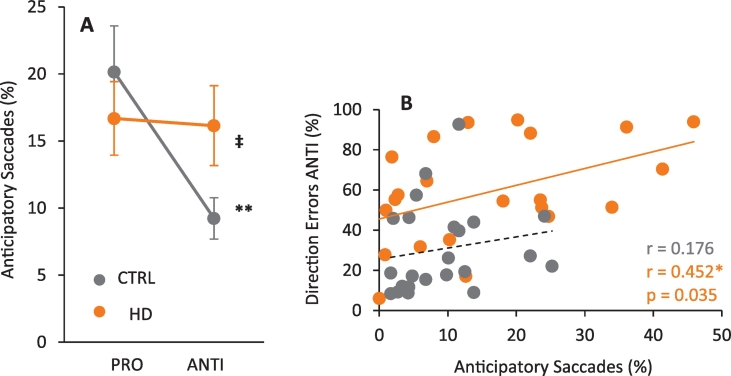
Anticipatory saccade behavior. A) Mean proportion of all (correct and erroneous) anticipatory saccades for both CTRL (dark grey) and HD (orange) groups. Error bars indicate SEM. (‡) *p* = 0.002, significance for group × task interaction only. (**) *p* = 0.001, significance for task interaction only. B) Pearson correlation between anticipatory saccades and direction error proportion in anti-saccade condition. (*) *p* = 0.035. (For interpretation of the references to color in this figure legend, the reader is referred to the online version of this chapter.)

The measurement of anticipatory saccades in the context of the pro- and anti-saccade task provides information about fixation control prior to the arrival of the visual stimulus information in oculomotor brain regions; thus it has been proposed that controlling anticipatory saccades could reflect voluntarily control mechanisms (Bell et al., 2006, Watanabe and Munoz, 2011b). We computed the mean proportion of anticipatory saccades (SRTs < 90 ms) for each participant for pro- and anti-saccade tasks. The analysis showed significant main effect of task (*F*_(1,43)_ = 13.879, *p* = 0.001, *η*^2^_*p*_ = 0.244), with a greater proportion of anticipatory saccades in the pro-saccade task (Fig. 4A). However, although there was no main effect of group (*F*_(1,43)_ = 0.225, *p* = 0.630, *η*^2^_*p*_ = 0.005), there was a group × task significant interaction (*F*_(1,43)_ = 11.402, *p* = 0.002, *η*^2^_*p*_ = 0.210) showing that HD patients made more anticipatory saccades in the anti-saccade task, compare to control subjects (see Table 2) (Fig. 4A). To know if there was a correlation between anticipatory anti-saccades and anti-saccade direction errors, we computed the two-tailed Pearson's correlation coefficient. The analysis showed a positive correlation for HD patients (*r*_(21)_ = 0.452, *p* = 0.035) only; the correlation for controls was not significant (*r*_(22)_ = 0.176, *p* = 0.423) (Fig. 4B).

### 3.2 Neural atrophy.

#### 3.2.1 Grey-matter atrophy

**Fig. 5:**
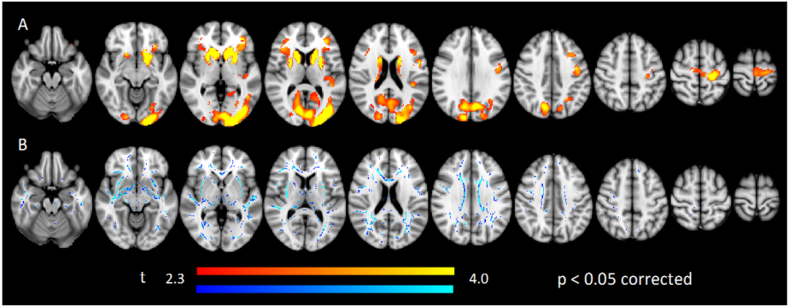
Axial sections showing areas where significant A) grey matter volume (red-yellow scale) and B) fractional anisotropy (blue-lightblue scale) reduction in HD disease compared with CTRL. Threshold set at *p* < 0.05, corrected. Images are set from inferior (left) to superior (right) views. The results are projected in MNI152 space. Left side of the image is right side of the brain (radiological representation). (For interpretation of the references to color in this figure legend, the reader is referred to the web version of this article.)

Voxel-based morphometry analyses were done using the high-resolution T1 weighted 3D images of the whole brain including brainstem and cerebellum to compare grey-matter volume of controls and HD patients. The regions with significantly less grey-matter volume (*p* < 0.05, corrected) in the HD patients are summarized in Fig. 5. The regions include: bilateral caudate and putamen nucleus; left premotor and precentral gyrus; inferior parietal lobe; right cuneus and left middle occipital gyrus. No regions were found where the grey-matter volume was larger in HD patients than in controls. A complete list of all the areas with significant differences is provided in Supplementary Table 1.

#### 3.2.2 White-matter atrophy

We used Tract-Based Spatial Statistics on FA maps from the whole brain, brainstem and cerebellum to compare between controls vs. HD. The regions with significant less white-matter FA (*p* < 0.05, corrected) in the HD patients are summarized Fig. 5. The regions include: bilateral external capsule; inferior fronto-occipital fasciculus; anterior and posterior corona radiata; cortico-spinal tract; superior longitudinal fasciculus and anterior thalamic radiation; right splenium of corpus callosum; forceps major; optic radiation and acoustic radiation; left inferior longitudinal fasciculus; posterior thalamic radiation; posterior optic radiation; cerebral peduncle; middle cerebellar peduncle and pontine crossing tract. No regions were found where the FA of water diffusion was larger in HD patients than in controls. A complete list of all the areas with significant differences is in Supplementary Table 2.

### 3.3 Relationship between saccade inhibition deficits and neural atrophy

#### 3.3.1 Grey-matter relationship

**Fig. 6:**
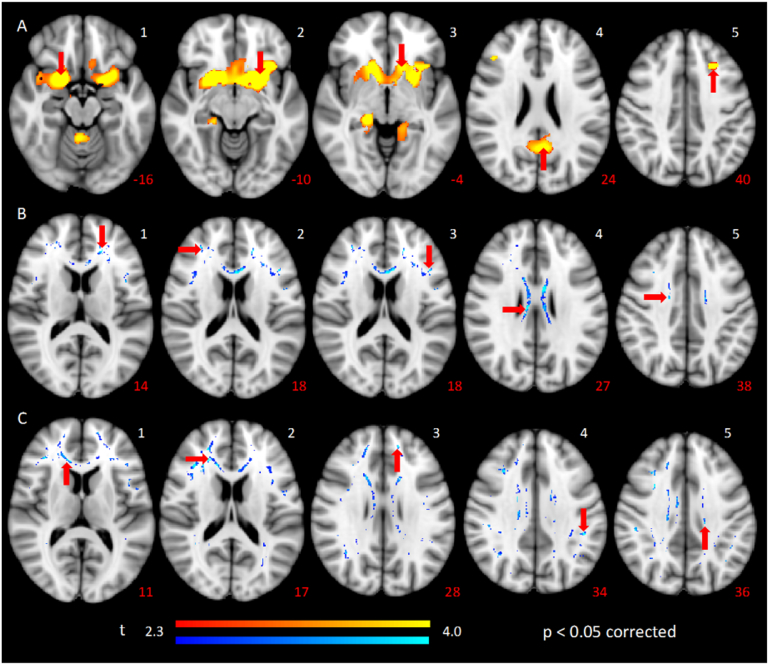
Axial sections showing areas where grey A) matter volume (red-yellow scale) and B, C) fractional anisotropy (blue-lightblue scale) reduction in HD patients correlate with saccade variables. A) Correlation between grey-matter and error proportion in regular-latency saccades anti-trials, 1: right frontal subcallosal gyrus. 2: left putamen. 3: left caudate head. 4: left parietal lobe, posterior cingulate, 5: right middle frontal gyrus. B) Correlation between fractional anisotropy and error proportion in regular-latency anti-saccades trials, B1: left anterior corona radiata. B2: right anterior thalamic radiation, frontal lobe. B3: left anterior thalamic radiation, frontal lobe B4: right cingulate gyrus, parietal lobe. B5: right superior corona radiata. C) Correlations between fractional anisotropy and proportion of anticipatory saccades anti-trials, C1: right anterior cingulate. C2: right superior corona radiata, frontal lobe. C3: left superior forceps minor, frontal lobe. C4: left superior longitudinal fasciculus, parietal lobe. C5: left superior corona radiata, parietal lobe. Threshold set at *p* < 0.05, corrected. White numbers indicate anatomical region. Red labels indicate the Z coordinates (in voxels). Red arrows indicate the main cluster of the anatomical region. Images are set from inferior (left) to superior (right) views. The results are projected in MNI152 space. Left side of the image is right side of the brain (radiological representation). Color bars represent the *t* value for the VBM (red-yellow) and TBSS (blue-lightblue) analyses. (For interpretation of the references to color in this figure legend, the reader is referred to the web version of this article.)

We analyzed whether the voluntary inhibitory deficits (anti-saccade errors and anticipatory anti-saccades) seen in HD patients could be explained by specific neural atrophy in circuits controlling eye movements (outside the indirect-pathway). We tested if there was a relationship (*p* < 0.05, corrected) between grey-matter volumes, white-matter values, and saccade variables. The grey-matter volumes showed negative relationships between regular-latency anti-saccade errors and left caudate and putamen nucleus; bilateral middle frontal gyrus; subcallosal gyrus; inferior frontal gyrus (Brodmann Area (BA) 47); left posterior cingulate (parietal lobe); right cerebellum and thalamus; right parahippocampal gyrus BA 27; temporal lobe BA 13 and left insula; Fig. 6A shows a summary of these areas and a complete list of all areas is provided in Table 3.

**Table 3:** Significant correlations between grey-matter and white-matter decrease with error proportion of regular-latency saccades anti-trials for HD patients.

Brain region	Anatomical location	Brodmann area	MNI coordinates			Cluster size mm^3^
			X	Y	Z	
Grey-matter						
Left cerebrum	Putamen	–	− 26	12	− 10	13
Left cerebrum	Caudate head	–	− 12	22	− 4	13
Right cerebrum	Thalamus	–	16	-24	0	9
Right cerebellum	Anterior lobe	–	4	− 48	− 20	6
Left parietal lobe	Posterior cingulate	–	− 4	− 56	24	10
Left parietal lobe	Parahippocampal gyrus	–	− 14	− 44	4	11
Right frontal lobe	Subcallosal gyrus	–	20	8	− 16	12
Left frontal lobe	Middle frontal gyrus	–	− 26	24	40	7
Right frontal lobe	Middle frontal gyrus	–	44	30	22	5
Left temporal lobe	Superior gyrus		− 36	4	− 26	13
Right temporal lobe	Parahippocampal gyrus	27	20	− 32	− 6	9
Right temporal lobe	Superior temporal gyrus	41	60	-20	8	8
Right temporal lobe	Temporal lobe	13	40	10	− 20	1
Left insula	Inferior frontal gyrus	47	− 34	20	− 8	13

White-matter						
Right frontal lobe	Inferior fronto-occipital fasciculus	–	34	33	12	82
Left frontal lobe	Inferior fronto-occipital fasciculus	–	− 28	32	− 5	77
Right frontal lobe	Anterior corona radiata, anterior thalamic radiation	–	23	33	17	73
Right frontal lobe	Superior gyrus	–	13	24	48	71
Left frontal lobe	Superior gyrus, forceps minor	–	− 14	46	28	42
Right parietal lobe	Forceps major	–	21	− 51	24	61
Left parietal lobe	Superior longitudinal fasciculus	–	− 45	− 37	34	65
Right parietal lobe	Precuneus	–	19	− 42	57	50
Left parietal lobe	Superior corona radiata, cingulate gyrus	–	− 18	− 24	36	32
Right cerebrum	Anterior cingulate, forceps minor	–	11	30	11	69
Right temporal lobe	Superior longitudinal fasciculus, posterior thalamic radiation	–	34	− 47	16	18
Left temporal lobe	Inferior fronto-occipital fasciculus	–	− 41	− 14	− 13	26

#### 3.3.2 White-matter relationship

The white-matter FA values showed negative relationships between regular-latency anti-saccade errors and bilateral inferior fronto-occipital fasciculus and anterior thalamic radiation; right corpus callosum, superior longitudinal fasciculus and superior corona radiata; left forceps minor and anterior corona radiata (Fig. 6B). A complete list of all the areas with significant differences is provided in Table 3. Also there was a negative relationship between proportion of anticipatory anti-saccades and bilateral inferior fronto-occipital fasciculus, superior longitudinal fasciculus and forceps minor; right anterior thalamic radiation, anterior corona radiata, forceps major and posterior thalamic radiation; and left superior corona radiata (Fig. 6C). A complete list of all the areas with significant differences is in Table 4.

**Table 4:** Significant correlation between white-matter decrease and proportion of anticipatory saccades anti-trials for HD patients.

Brain region	Anatomical location	MNI coordinates			Cluster size mm^3^
		X	Y	Z	
Right frontal lobe	Inferior fronto-occipital fasciculus	34	33	12	82
Left frontal lobe	Inferior fronto-occipital fasciculus	− 28	32	− 5	77
Right frontal lobe	Anterior corona radiata, anterior thalamic radiation	23	33	17	73
Right frontal lobe	Superior gyrus	13	24	48	71
Left frontal lobe	Superior gyrus, forceps minor	− 14	46	28	42
Right parietal lobe	Forceps major	21	− 51	24	61
Left parietal lobe	Superior longitudinal fasciculus	− 45	− 37	34	65
Right parietal lobe	Precuneus	19	− 42	57	50
Left parietal lobe	Superior corona radiata, cingulate gyrus	− 18	− 24	36	32
Right cerebrum	Anterior cingulate, forceps minor	11	30	11	69
Right temporal lobe	Superior longitudinal fasciculus, posterior thalamic radiation	34	− 47	16	18
Left temporal lobe	Inferior fronto-occipital fasciculus	− 41	− 14	− 13	26

## 4 Discussion

The aim of this study was to evaluate if atrophy in structures outside the indirect-pathway of the basal ganglia were related to voluntary saccade inhibitory deficits in HD patients. Our behavioral findings in HD patients showed a positive correlation between anti-saccade direction errors and anticipatory anti-saccades. The imaging analyses showed extensive cortico-basal ganglia circuitry atrophy. Notably, there were positive relationships between regionally grey and white-matter atrophy in thalamic, frontal, parietal and cingulate regions with the percentage of anti-saccade errors and anticipatory anti-saccades. These results suggest that deficits in the implementation of voluntary saccade inhibition control are related to atrophy in a neural network that extends well beyond the indirect-pathway within the basal ganglia. We discuss our findings in relation to the neural circuitry controlling voluntary saccades and the neural atrophy in HD.

### 4.1 Anti-saccade direction errors

Our results showed that HD patients made more anti-saccade errors than controls and this was driven by an increase in the number of errors in longer latency anti-saccades, but not by express saccades. The occurrence of these errors was positively correlated with neurodegeneration in the voluntary control saccade system including grey-matter atrophy in caudate and putamen nucleus, thalamic, frontal, parietal, and cingulate regions. In addition, we also found relationships with white-matter atrophy in the superior longitudinal fasciculus, anterior thalamic radiation and anterior corona radiata that interconnect the areas described above.

An influential basal ganglia model initially suggested that atrophy in the indirect-pathway (mainly the caudate nucleus) in HD, leads to a tonic over-activation in the neural network controlling saccadic eye movements (Albin et al., 1990, Anderson and MacAskill, 2013, DeLong, 1990, Ross and Tabrizi, 2011, Sharp and Ross, 1996). Thus, the voluntary saccade inhibition deficits in HD patients were attributed to such atrophy (Anderson and MacAskill, 2013, Walker, 2007). However, recent evidence suggests that atrophy could include motor cortex, supplementary motor area, premotor cortex, cingulate cortex, dorsolateral prefrontal cortex and subthalamic nucleus, which are collectively termed the hyperdirect pathway; the atrophy of this pathway in HD increases substantia nigra reticulata/globus pallidus activity and suppresses movements by strengthening the inhibition of thalamocortical activity (Hikosaka et al., 2006, Nambu et al., 2002). Neurocomputational modelling also suggests that combined lesions of the direct/indirect pathways, but not lesion of either pathway alone explains the motor, learning, and inhibitory deficits in HD patients (Schroll et al., 2015). This concept is also supported by the therapeutic effect of pallidotomy in hyperkinetic disorders (Okun and Vitek, 2004, Zhuang et al., 2009) and from imaging studies showing correlations between executive-motor deficits and premotor, sensorimotor (Douaud et al., 2006, Rosas et al., 2008), thalamic (Kassubek et al., 2005), substantia nigra and pallidal atrophy in HD (Douaud et al., 2006).

Our imaging results showed that anti-saccade errors were related to cortico-basal ganglia circuit atrophy, including grey-matter degeneration in caudate and putamen, thalamus, cingulate gyrus, parietal cortex and middle frontal gyrus. Anti-saccade errors were also correlated with white-matter atrophy in the superior longitudinal fasciculus, anterior thalamic radiation and anterior corona radiata. These results are supported by previous work showing correlations between anti-saccade errors with bilateral striatum and external/internal capsule atrophy (Scahill et al., 2013). Notably, it has been suggested that these fasciculi contribute to the hyperdirect-pathway that conveys projections from motor, supplementary motor, premotor and cingulate cortices to the subthalamic nucleus to exert a synergistic effect along with the indirect-pathway to suppress movements (Jahanshahi et al., 2015, Lehéricy et al., 2004). The observed degeneration pattern is congruent with the behavioral deficits we observed because anti-saccade errors are related to frontal eye field atrophy (Guitton et al., 1985). Taken together, these findings suggest that the voluntary saccade inhibition deficits seen in HD are related to the disruption of an extended network beyond the indirect basal ganglia pathway, including putamen; thalamus; cingulate gyrus; parietal and frontal cortices; as well as the longitudinal fasciculus, the thalamic radiation and the corona radiata.

### 4.2 Anticipatory saccade behavior

HD patients made more anticipatory saccades than controls. The occurrence of these anticipatory saccades had a positive correlation with anti-saccade errors, together suggesting reduced inhibitory control. Our imaging analysis showed critical positive relationships between white-matter atrophy, and the percentage of anticipatory anti-saccades in the inferior fronto-occipital fasciculus, anterior thalamic radiation, anterior corona radiata, forceps major and superior longitudinal fasciculus.

The significant increase of anticipatory saccades in HD patients performing the pro- and anti-saccade task had not been reported previously. Anticipatory saccade behavior can be triggered prior to the arrival of the visual stimulus information to the oculomotor system when saccade preparation signals are high (Bell et al., 2006, Dorris and Munoz, 1998, Marino et al., 2012). In the context of the pro- and anti-saccade task, these saccades have been linked to caudate nucleus' activity prior to the target stimulus appearance; this activity in turn releases superior colliculus and frontal eye field outputs facilitating anticipatory saccade behavior (Watanabe and Munoz, 2011b, Yang et al., 2008). Thus, it has been suggested that controlling anticipatory saccades could reflect general mechanisms involved in voluntary control (Watanabe and Munoz, 2011b).

Performing correct anti-saccades trials requires the ability to execute an incongruent stimulus-response mapping where an automatic response is inhibited and the correct response is prepared in the opposite hemisphere and then executed (Munoz and Everling, 2004). In order to properly execute this incongruent mapping, and before the appearance of the peripheral target, the brain must establish a preparatory set to execute the appropriate action (Connolly et al., 2002, Everling and Munoz, 2000). For example, patients with response inhibition problems involving prefrontal cortices and basal ganglia like obsessive-compulsive disorder, show a high proportion of anticipatory saccades manifested as saccade intrusions (perseverative behavior) or direction errors in oculomotor tasks (Spengler et al., 2006). Thus, the high anticipatory saccade behavior in HD patients could reflect impaired preparatory set activity hindering the vector inversion to transforming the initial location of the target into the appropriate motor command for the saccade execution (Munoz and Everling, 2004, Watanabe and Munoz, 2011a), resulting in an increase of longer latency direction errors (Coe and Munoz, 2017). The control group behavior supports this idea. They had a low proportion of anticipatory anti-saccades, likely because they could inhibit anticipatory saccades, resulting in a lack of correlation between anticipatory anti-saccades and regular-latency anti-saccade errors.

The imaging analysis showed relationships between the percentage of anticipatory anti-saccades and white-matter atrophy in the inferior fronto-occipital fasciculus, anterior thalamic radiation, anterior corona radiata (frontal lobe), forceps major (anterior cingulate), and superior longitudinal fasciculus (parietal lobe). It should be noted that regular-latency anti-saccade errors showed significant correlations with the same fasciculi that, as discussed above, are part of the neural network for inhibition in the cortico-basal ganglia circuitry (Jahanshahi et al., 2015, Lehéricy et al., 2004). These findings are supported by the proposal that the pathogenesis in HD begins in the myelin even before the appearance of motor and cognitive impairments (Bartzokis et al., 2007, Ehrlich, 2012). Myelin abnormalities can slow fast axon transport resulting in synaptic loss and eventually axonal degeneration (Han et al., 2010). Therefore, early white-matter atrophy could disrupt the cortico-basal ganglia circuitry, which affects the preparatory neural activity required to establish preparatory set and therefore impacts the ability to execute appropriate saccades in early HD patients resulting in both types of behavioral deficits (Connolly et al., 2002, Everling and Munoz, 2000, Klöppel et al., 2008). Other studies support the idea of early HD's white-matter effect on executive deficits, suggesting that in vivo measures of HD's white-matter can be a reliable marker of cognitive deficits progression (Matsui et al., 2015).

### 4.3 Conclusions

Our findings showed a significant increase in anti-saccade direction errors and anticipatory anti-saccades in early HD patients. Furthermore, the regular-latency anti-saccade errors and anticipatory anti-saccades, which correlated with each other, showed specific relationship with regionally white-matter atrophy in the inferior fronto-occipital fasciculus, anterior thalamic radiation, anterior corona radiata, forceps major and superior longitudinal fasciculus. These fasciculi contribute to the hyperdirect-pathway conveying projections from motor, supplementary motor, premotor and cingulate cortices to the subthalamic nucleus to exert a synergistic effect along with the indirect-pathway to suppress movements. These results suggest that impairments in the implementation of voluntary inhibitory behaviors could be explained by early myelin atrophy in the cortico-basal ganglia circuitry, which in turn impairs the establishment of the preparatory set activity in the neural network controlling voluntary eye movements for the execution of the appropriate saccade. Further research combining behavioral measures and MRI techniques should explore the neural basis of voluntary saccade inhibitory deficits as a possible marker of cognitive deficits progression in HD.

## Appendix A Supplementary data

Supplementary material Image 1
