# Supplementary material for: Voluntary saccade inhibition deficits correlate with extended white-matter cortico-basal atrophy in Huntington's disease
Source: Neuroimage Clin. 2017 Jun 9;15:502–12. doi: 10.1016/j.nicl.2017.06.007 (PMC5472191; doi:10.1016/j.nicl.2017.06.007)
Supplement: Supplementary file 1 — Supplementary material [file mmc1.docx]

**Supplementary Material**

| **Supplementary Table 1**. Neuroanatomical regions showing a significant decrease of grey-matter volume in HD patients | | | | | | |
| --- | --- | --- | --- | --- | --- | --- |
| Brain Region | Anatomical Location | Brodmann Area | MNI coordinates | | | Cluster size mm^3^ |
|  |  |  | X | Y | Z |  |
| Right cerebrum | Caudate | - | 6 | 22 | 6 | 10 |
| Left cerebrum | Caudate | - | -8 | 10 | 8 | 11 |
| Right cerebrum | Putamen | - | 24 | 4 | 12 | 10 |
| Left cerebrum | Putamen | - | -24 | 6 | 12 | 11 |
| Left frontal lobe | Premotor cortex | 6 | -14 | -24 | 62 | 9 |
| Left frontal lobe | Precentral gyrus | 44 | -48 | 0 | 22 | 4 |
| Left parietal lobe | Inferior | - | -40 | -38 | 16 | 2 |
| Right occipital lobe | Cuneus | 17 | 10 | -80 | 8 | 12 |
| Left occipital lobe | Middle occipital gyrus | 18 | -24 | -92 | 10 | 12 |

**Supplementary Table 1**. Coordinates represent the peak value of the cluster and are in the MNI152 space in mm. Correspondence with Brodmann areas was obtained through Talairach Daemon.

| **Supplementary Table 2**. Neuroanatomical regions showing a significant decrease of white-matter volume in HD patients | | | | | |
| --- | --- | --- | --- | --- | --- |
| Brain Region | Anatomical Location | MNI coordinates | | | Cluster size mm^3^ |
|  |  | X | Y | Z |  |
| Right cerebrum | Putamen, external capsule, superior longitudinal fasciculus | 32 | -9 | 9 | 482 |
| Left cerebrum | Putamen, external capsule, superior longitudinal fasciculus | -32 | 0 | 8 | 480 |
| Left cerebrum | Putamen, external capsule, inferior occipito-frontal fascicle | -30 | 8 | -8 | 466 |
| Right cerebrum | External capsule, inferior fronto-occipital fasciculus | 35 | -7 | -10 | 472 |
| Right cerebrum | Anterior thalamic radiation | 9 | -21 | 13 | 450 |
| Left cerebrum | Anterior thalamic radiation | -3 | -8 | 10 | 412 |
| Left cerebrum | Thalamus, posterior limb of internal capsule, cortico-spinal tract | -22 | -19 | 1 | 227 |
| Right cerebrum | Brainstem, midbrain | 19 | -20 | 4 | 398 |
| Left cerebrum | Brainstem, cerebral peduncle, cortico-spinal tract | -12 | -17 | -9 | 302 |
| Left cerebrum | Brainstem, pons, pontine crossing tract | -4 | -33 | -36 | 217 |
| Left cerebrum | Brainstem, pons, middle cerebellar peduncle | -14 | -24 | -30 | 210 |
| Right cerebrum | Frontal lobe, anterior corona radiate | 19 | 27 | 21 | 481 |
| Right cerebrum | Frontal lobe, anterior corona radiate | 17 | 37 | 2 | 434 |
| Left cerebrum | Frontal lobe, anterior corona radiata, forceps minor | -17 | 26 | 23 | 473 |
| Right cerebrum | Posterior corona radiata, inferior fronto-occipital fasciculus | 29 | -46 | 22 | 479 |
| Left cerebrum | Posterior corona radiata | -20 | -26 | 32 | 477 |
| Right frontal lobe | Anterior cingulate, uncinate fasciculus | 12 | 35 | -13 | 445 |
| Right frontal lobe | Inferior frontal gyrus, inferior fronto-occipital fasciculus | 39 | 29 | -5 | 441 |
| Left frontal lobe | Inferior frontal gyrus, uncinate fasciculus | -30 | 26 | -14 | 436 |
| Left frontal lobe | Precentral gyrus, superior longitudinal fasciculus | -39 | -9 | 29 | 474 |
| Right parietal lobe | Splenium of corpus callosum, forceps major | 25 | -53 | 12 | 479 |
| Right parietal lobe | Cingulate gyrus, callosal body | 6 | -13 | 27 | 475 |
| Left parietal lobe | Posterior thalamic radiation, optic radiation | -26 | -63 | 13 | 478 |
| Left parietal lobe | Superior longitudinal fasciculus | -38 | -43 | 25 | 467 |
| Right cerebrum | Anterior cingulate, frontal lobe | 10 | 26 | 21 | 453 |
| Right cerebrum | Cingulate gyrus, parietal lobe, splenium of corpus callosum | 17 | -36 | 32 | 440 |
| Left cerebrum | Cingulate gyrus, callosal body | -16 | -24 | 31 | 447 |
| Left cerebrum | Anterior cingulate | -12 | 19 | 22 | 473 |
| Left temporal lobe | Superior temporal gyrus, inferior longitudinal fasciculus | -45 | -24 | -2 | 471 |
| Left temporal lobe | Posteriro thalamic radiation, inferior fronto-occipital fasciculus | -29 | -49 | 18 | 470 |
| Right temporal lobe | Inferior fronto-occipital fasciculus | 41 | -29 | -9 | 464 |
| Left temporal lobe | Fusiform gyrus | -37 | -42 | -16 | 431 |
| Right temporal lobe | Acoustic radiation | 48 | -12 | -5 | 430 |
| Left temporal lobe | Parahippocampal gyrus | -32 | -2 | -24 | 393 |
| Left occipital lobe | Optic radiation, forceps major | -24 | -77 | 1 | 421 |
| Right occipital lobe | Middle occipital gyrus | 39 | -71 | 8 | 419 |

**Supplemntary Table 2**. Coordinates represent the peak value of the cluster and are in the MNI152 space in mm. Anatomic region labels are from the Johns Hopkins University white matter atlas.

Algorithm design description

1. Blinks detection and removal. Blinks are detected using the loss of the eye tracking signal from the eye tracker and then filled in with linearly interpolated data.
2. Position data converted from pixels to degrees of visual angle and centered to the screen. We used the mode of the fixation position (position from 500ms before fixation point disappearance to fixation point disappearance) for each block of 40 trials to correct drift data position.
3. Velocity was calculated using a three point sliding window in the X and Y, smoothed using a three point rectangular filter, and then combined in to speed.
4. Saccades were detected by calculating a speed threshold. This is calculated by finding the mode of the fixation speed signal per trial. Fixation per trial is defined as any period where the speed is less than 50 degrees/s and there is no blink. The mode of the fixation + 2* the standard deviation of the fixation is the saccade speed threshold. Saccades are then detected as any time the speed is above this threshold for five more consecutive sample points. Metrics are then calculated on these saccades for analysis.
5. Each trial is then automarked to parse out correct and incorrect eye movements. Trials with excessive loss of eye tracking or large drift (4 degree wide window around fixation) are discarded.
